# Beyond Andromeda: Improving Therapy for Light Chain Amyloidosis

**DOI:** 10.3389/fonc.2020.624573

**Published:** 2021-02-03

**Authors:** Gregory P. Kaufman, Claudio Cerchione

**Affiliations:** ^1^ Department of Lymphoma & Myeloma, The University of Texas MD Anderson Cancer Center, Houston, TX, United States; ^2^ Hematology Unit, Istituto Scientifico Romagnolo per lo Studio e la Cura dei Tumori (IRST) IRCCS, Meldola, Italy

**Keywords:** amyloidosis, daratumumab, NEOD001, CAEL-101, BCMA, CD-38

## Abstract

Therapy for light chain amyloidosis (AL) continues to evolve, and a new standard of care for the disease is rapidly forming. The risk of early death however, mainly from cardiac complications, remains an important benchmark yet to be definitively improved upon. This brief review explores recent advances in plasma cell directed therapy for AL, highlighting unique factors specific to these patients and AL biology driving differences in treatment strategies and clinical development compared with multiple myeloma. Improving upon proteasome inhibitor based upfront therapy combinations with the addition of anti-CD38 antibodies has shown promise with improved response rates in the ANDROMEDA (NCT03201965) study. Though depth and kinetics of achieving deep hematologic response as well as rates of biomarker defined organ response were improved with the addition of daratumumab to the combination of bortezomib, cyclophosphamide, and dexamethasone, death rates in each arm remained similar. Evaluation of other targeted and novel therapies in AL is ongoing, and we highlight efforts evaluating B-cell maturation antigen (BCMA) directed therapy, BCL-2 family inhibitors, and other novel agents in the field. We also look ahead to efforts to reimagine the clinical development of anti-fibrillar therapies after late phase study failures. Upcoming anti-amyloid fibril antibody studies explore opportunities to improve outcomes for the sickest AL patients with advanced cardiac disease, focusing on improving overall patient survival and reducing the risk of early death in this uniquely frail population.

## Introduction and Brief Review

Progressive organ dysfunction driven by amyloid deposition and risk of early death is a hallmark of AL amyloidosis, particularly for those with cardiac involvement, which includes the majority of patients (~70%) (1). Plasma cell directed therapy has improved outcomes for these patients given the intrinsic linkage between hematologic response rates, organ response rates, and survival (2). Oft quoted is the poor prognosis of advanced cardiac amyloid nearing 4 months in advanced involvement, though studies have shown improved survival with successfully achieving hematologic response endpoints (3, 4). Still, selection bias and clinical status have often excluded many advanced cardiac patients from prospective clinical trials and some retrospective series. Manwani et al. reported in a retrospective series of 915 patients with AL treated with upfront bortezomib, 51% percent of whom had stage III cardiac disease, a complete hematologic response rate of 15%, and a cardiac organ response rate of 32% (4). These results were similar to an upfront study of CyBorD in stage III cardiac AL, which did of note include patients with baseline NT-proBNP greater than 8,500 ng/L, that demonstrated a 40% VGPR or CR response rate and 1 year overall survival of 57% for this high risk cohort, though notably found marked inferior survival outcomes among the 40% of patients with baseline NT-proBNP >9,500 ng/L (5). Based on these and similar findings, bortezomib based upfront therapy, primarily the CyBorD combination, has become a highly used standard of care for the initial treatment of systemic AL (6). Of note, given the risk of worsening neuropathy in this population, the oral proteasome inhibitor ixazomib has been evaluated in the TOURMALINE-AL1 study of relapsed AL with encouraging secondary endpoints favoring ixazomib–dexamethasone over physician’s choice combinations in terms of patient survival and preservation of organ function, although hematologic response rates were similar (7). Ixazomib is currently being evaluated in a phase II study in combination with cyclophosphamide and dexamethasone for treatment of naïve AL, with early results demonstrating 57% hematologic overall response rates including 26% VGPR and 14% amyloid CR (8).

High dose melphalan and autologous stem cell transplantation in appropriately selected AL patients can achieve excellent long term disease control, though often advanced cardiac AL will preclude eligibility evaluation for transplant unless upfront therapy enables clinical improvement (9). Sequential CyBorD induction and proceeding to stem cell transplant for patients with an unsatisfactory response is a proven treatment strategy, though often driven by the benefit for patients without stage III cardiac disease (10–12). A multicenter retrospective cohort of 22 patients (86% of whom were stage III with regard to cardiac status at diagnosis) has shown the feasibility of deferred stem cell transplantation, either for consolidation or relapse, in patients initially deemed transplant ineligible due to cardiac status (13). While this cohort demonstrates the feasibility of improving patients’ clinical status with successful upfront bortezomib based therapy, overall hematologic CR rates for patients with revised Mayo stage IIIA or IIIB cardiac amyloid treated with CyBorD plateau at around 14–23% (6). Combined with the knowledge of data showing that around 80% of patients obtaining a hematologic CR will reach the threshold of a cardiac organ response, the majority of advanced cardiac AL patients have significant room for improvement in outcomes (2).

## Daratumumab Emerges as a Treatment

Daratumumab has demonstrated responses of high clinical interest in retrospective and prospective reported studies in heavily pretreated AL patients with reported rates of VGPR/CR of 47–86% (14–16). These encouraging hematologic response rates, many of which were achieved in patients who had never previously achieved a deep level of free light chain control to prior line therapies, resulted in cardiac organ response rates of 50–55% (16, 17). The randomized prospective ANDROMEDA study (NCT03201965) aims to show the potential improvement in hematologic response rates with the addition of subcutaneous daratumumab to CyBord and how this will translate to meaningful improvement in long term patient outcomes in the newly diagnosed AL setting. Of note patients with NT-proBNP greater than 8,500 ng/L were not eligible for ANDROMEDA, though 71% of patients had cardiac involvement with 37% of all patients having stage III disease (18, 19). Primary results from ANDROMEDA have as of now been reported at the 2020 EHA meeting. With the use of subcutaneous daratumumab, systemic administration reactions were low (4% of patients with grade 1 cough and hypotension), and the need for intravenous fluids with intravenous daratumumab was avoided in this potentially volume sensitive cardiac population. Patients randomized to the daratumumab combination arm had significantly longer median duration on treatment for their initial therapy (9.6 *vs* 5.3 months), with only 9.7% of Dara-CyBorD randomized patients receiving subsequent therapy compared to 41% of patients in the CyBorD alone arm (with 60% of these patients effectively crossing over to receive daratumumab). Overall hematologic response rates as well as VGPR/CR rates both significantly favored the Dara-CyBorD arm (ORR 92 *vs* 77%; VGPR/CR 79 *vs* 49%). These results support the use of Dara-CyBorD as upfront therapy in systemic AL and demonstrate the ability to achieve a VGPR/CR for the majority of patients for the first time. The composite time to event endpoint of progression free survival and major organ deterioration also favored the Dara-CyBorD combination (HR 0.58; CI 0.36–0.93, P = 0.022). Cardiac organ response rates at six months favored the addition of the anti-CD38 antibody as well, with the near doubling of patients achieving a cardiac response (42 *vs* 22%). Notable side effects included a low rate (7%) of grade 1–2 administration-related reactions with the use of subcutaneous daratumumab as well as slightly higher rates of pneumonia (8 *vs* 4%), lymphocytopenia (13 *vs* 10%), and diarrhea (6 *vs* 4%) with the addition of daratumumab. Still, despite the significant improvement in response parameters and overall tolerability with the addition of daratumumab to the CyBorD backbone, patient deaths were relatively equal between the two arms (27 patients in the Dara-CyBorD arm and 29 patients in the CyBorD arm) (18). Additional questions remain about the specific outcomes of the subgroup of patients with stage IIIA cardiac AL, as well as to the generalizability to patients with Stage IIIB disease. Several additional studies are ongoing in the upfront setting for patients with advanced cardiac AL. These include a study of daratumumab monotherapy in patients with Stage IIIB AL (NCT04131309) being conducted primarily in Europe, the combination of daratumumab, bortezomib, and dexamethasone in revised Mayo stage IV patients (NCT04474938), as well as a study of daratumumab, ixazomib, and dexamethasone for both newly diagnosed and previously treated systemic AL patients (NCT03283917). While the addition of the anti-CD38 antibody daratumumab to CyBorD clearly improves surrogate response endpoints, such as depth of hematologic response, in newly diagnosed systemic AL, its exact role in upfront therapy and patient selection for alternative therapies including high dose melphalan and autologous stem cell transplant remain the central questions to improve outcomes for many AL patients.

## Additional Therapy Opportunities in AL

Alkylating agents have long held a central role in the therapy of systemic AL. Both cyclophosphamide based combination therapy and melphalan have been used extensively in the early treatment of patients (20). The question of whether cyclophosphamide adds value and improves survival when added to bortezomib and dexamethasone for the upfront treatment of AL has recently been explored with multiple evaluations showing no clear benefit with respect to survival or depth of hematologic and organ response rates (21, 22). Other alkylating therapies are being explored in the previously treated AL setting. Bendamustine is an alkylating agent that has been evaluated in previously treated AL with modest results, with an overall hematologic response rate of 32–57%, and only 12–29% of patients achieving an organ response (23, 24). Melphalan Flufenamide (Melflufen) is a novel peptide-drug conjugate that is metabolized inside malignant plasma cells to melphalan, and in the setting of previously treated multiple myeloma has shown efficacy even in alkylator resistant cases (25, 26). Evaluation of Melhalan Flufenamide in combination with dexamethasone in patients with previously treated AL is currently being evaluated (NCT04115956). Given the historical success of delivering high dose melphalan to the relatively indolent plasma cell neoplasm underlying AL, as compared with multiple myeloma, with long term survival in AL patients being a well described experience, there is high excitement about the potential for melflufen in this setting (27).

The large structural chromosomal abnormality t(11;14) is overrepresented in the plasma cell neoplasm underlying AL compared with multiple myeloma, with about 50% of AL pati ents showing t(11;14) positivity compared to about 15% in newly diagnosed multiple myeloma (28–31). Patients with AL amyloidosis and t(11;14) have been found to have inferior hematologic and organ response rates as well as inferior five-year overall survival compared to non-t(11;14) AL patients in the setting of upfront bortezomib based therapies (32). The presence of t(11;14) is associated with a high BCL-2/MCL-1 ratio and confers sensitivity to venetoclax in clinical studies of relapsed or refractory multiple myeloma (33, 34). Given this, there has been interest in venetoclax and other BCL-2 family inhibitory compounds for plasma cell directed therapy in AL. Given the availability of venetoclax for off-label use, several retrospective case reports and series have been reported showing high hematologic response rates and general tolerability (35). Reported hematologic response rates in t(11;14) AL have been high, with Sidiqi et al. reporting seven of eight evaluable patients achieving either a CR or VGPR among a heavily pretreated population; additionally venetoclax at a dose of either 400 mg or 800 mg daily was generally well tolerated with the majority of patients experiencing low grade GI toxicity. As this was a small retrospective reported series in which venetoclax was used in varying therapy combinations, it is difficult to generalize when speaking of response rates, though ongoing planned studies of venetoclax and other BCL-2 targeted agents are planned in both previously treated and newly diagnosed AL harboring t(11;14).

B-cell maturation antigen (BCMA) is overexpressed on the surface of neoplastic plasma cells, and has been validated as a target in the treatment of multiple myeloma (36, 37). Likewise, early studies have shown preservation of high membrane bound BCMA expression on the clonal plasma cells underlying AL (38, 39). One study of bone marrow specimens in 28 patients with AL amyloidosis demonstrated universal plasma cell BCMA expression of >50% with a median of 65% (50–80) (39). Other studies have shown that like multiple myeloma, gamma secretase inhibitors can increase membrane bound BCMA expression on clonal plasma cells in AL. In one study the gamma secretase inhibitor LY-411575 increased both mBCMA expression on ALMC-1 cells *in vitro* (mBCMA expression increased from 84 to 99%), as well as on CD138 selected cells from bone marrow aspirates of AL patients from 36 to 68% (40). Soluble BCMA has been shown to correlate with disease activity and may play a role in mediating resistance to BCMA targeted therapies; as such the relatively lower burden of clonal plasma cells in AL may represent a therapeutic opportunity for BCMA targeted therapies. Therapies targeting BCMA are undergoing extensive development in the myeloma field, and as of late 2020 belantamab mafodotin, an antibody drug conjugate, has been FDA approved for the treatment of relapsed multiple myeloma. Development of antibody drug conjugates, bispecific antibodies, and T cell engager therapies as well as CAR-T cell therapies are under consideration and are in the early stages at this time.

While plasma cell directed therapies have shown the ability to induce hematologic and organ responses that correlate with improvements in patient survival, the ability to directly target soluble and deposited amyloidogenic light chains and fibrils would present an attractive opportunity to uniquely target AL pathogenesis. NEOD001, also known as birtamimab, was developed based on the binding to an epitope of serum amyloid A protein, though it also demonstrated reactivity to AL amyloid extracts and fibrils consisting of light chain immunoglobulins (41). In a phase 1/2 study of NEOD001 conducted in patients with persistent organ dysfunction following plasma cell directed therapy, 57% of eligible patients achieved an NT-proBNP defined cardiac organ response, and 60% met the threshold for renal response (42, 43). While these organ response rates were seen as encouraging in relation to expectations with historically treated patients of a similar population, questions remained about the clinical significance of biomarker defined organ responses and a randomized phase III study with a primary endpoint containing survival outcomes was planned to evaluate efficacy. The VITAL study randomized patients with treatment naïve AL with cardiac dysfunction to standard of care CyBorD as plasma cell directed therapy with or without NEOD001, with a primary composite endpoint of all-cause mortality or cardiac hospitalization (44). The study was terminated early after an interim futility analysis showed lack of efficacy and risk of increased harm in the cohort receiving NEOD001. However, a *post hoc* analysis focusing on advanced cardiac AL patients, looking at overall survival without regard to cardiac hospitalizations, showed potential benefit with a hazard ratio of 0.498 (95% CI 0.24–1.03) P = 0.055, among 77 Mayo stage IV patients (45). Based on these results, further studies in advanced cardiac AL focusing on overall survival are planned. CAEL-101 is another promising anti-light chain antibody in development with phase I and II studies showing encouraging results. In dose escalation and expansion cohorts, CAEL-101 showed no high grade toxicity and demonstrated rapid organ responses in 60–80% of patients (46). Follow-up data confirms 78% of patients are alive at 37 months of median follow-up, with high rates of sustained organ response (47). Based on these and confirmatory phase II dosing studies, twin randomized phase III studies in treatment naïve Mayo stage IIIA or IIIB cardiac AL patients are being conducted (NCT04512235 and NCT04504825).

## Discussion

Therapy for light chain amyloidosis is rapidly evolving, and due to distinct disease pathophysiology and biology, the paradigm of developing combination therapies and sequences is further diverging from multiple myeloma. Upcoming trials evaluating anti-CD38 monoclonal antibody combinations, BCMA and BCL-2 family targeting agents, anti-amyloid fibril targeting antibodies, and other novel therapies are poised to generate a new standard of care, so urgently needed in this devastating disease.

## Author Contributions

GK and CC conceptualized and wrote the article. All authors contributed to the article and approved the submitted version.

## Conflict of Interest

GK receives research funding for the conduct of clinical trials from Janssen, BMS, and Caelum Biosciences.

The remaining author declares that the research was conducted in the absence of any commercial or financial relationships that could be construed as a potential conflict of interest.
